# Hepatitis C services at harm reduction centres in the European Union: a 28-country survey

**DOI:** 10.1186/s12954-019-0290-x

**Published:** 2019-03-21

**Authors:** Juan M. Pericàs, Daniel J. Bromberg, Denise Ocampo, Eberhard Schatz, Iwona Wawer, Piotr Wysocki, Kelly Safreed-Harmon, Jeffrey V. Lazarus

**Affiliations:** 1 Barcelona Institute for Global Health (ISGlobal), Hospital Clínic, University of Barcelona, Carrer Rosselló, 132, 4th floor, ES-08036 Barcelona, Spain; 2 Correlation Network, De Regenboog Groep, Amsterdam, The Netherlands; 3 National AIDS Centre, Ministry of Health of Poland, Warsaw, Poland; 4 CHIP, Rigshospitalet, University of Copenhagen, Copenhagen, Denmark

**Keywords:** Hepatitis C, Cascade of care, Direct-acting antivirals, European Union, Survey

## Abstract

**Background:**

In the context of the WHO’s 2016 Viral Hepatitis Strategy and the introduction of treatment that can cure more than 95% of cases with hepatitis C virus (HCV) infection, the European Joint Action on HIV and Co-infection Prevention and Harm Reduction (HA-REACT) undertook a study in the member states of the European Union (EU). It aimed to determine service providers’ understanding of the current services in their respective countries and the barriers experienced by PWID in accessing HCV testing, care and treatment services in their country.

**Methods:**

In 2017, 38 purposively selected harm reduction service providers completed a 26-item English-language online survey addressing the availability, accessibility and funding of HCV services at harm reduction centres. HCV-related data and reported findings were extracted by country or by responding organization.

**Results:**

Responses were received from all EU member states. Respondents from 23 countries reported that HCV tests are offered by harm reduction services in their countries, and eight countries reported that addiction specialists in their countries are able to prescribe HCV therapy. Almost half of the respondents (45%) said that their respective organizations had established referral systems with centres providing HCV treatment.

**Conclusions:**

Not all EU member states have harm reduction services that provide HCV tests, and many do not have established referral systems with treatment providers. Moreover, the inability of addiction specialists to prescribe HCV treatment points to missed opportunities to make treatment more accessible. Further, discrepancies were noted between the available HCV services and stakeholders’ knowledge about their availability.

## Key points

There are important gaps in the HCV cascade of care in harm reduction services in several EU member states and discrepancies between the availability of these services and stakeholders’ knowledge. These include testing provision, referral systems and prescription of HCV treatment by addiction specialists.

## Introduction

An estimated 15.6 million people injected illicit drugs worldwide in 2017 [1]. In the absence of appropriate harm reduction measures, people who inject drugs (PWID) are disproportionately impacted by infectious diseases, viral infections among them. Today, injecting drug use remains the world’s single largest risk factor for the transmission of hepatitis C virus (HCV) [2], an epidemic that has chronically infected an estimated 71 million people as of 2015, accounting for approximately 1% of the world’s population [3, 4]. Left untreated, infection with HCV can lead to cirrhosis, liver failure and hepatocellular carcinoma [4]. The rate of progression and time to liver fibrosis and related complications are similar among PWID and HIV-uninfected people living with HCV [5].

The growing recognition of the global burden of hepatitis has led to the emergence of a recent, internationally coordinated response. Major international organizations, including the European Monitoring Centre for Drugs and Drug Addiction (EMCDDA) [6], European Centre for Disease Prevention and Control (ECDC) [7] and World Health Organization (WHO) [8], recommend that prevention, vaccination, diagnosis and treatment of viral hepatitis be available to PWID as part of a comprehensive harm reduction package. In 2016, WHO published its landmark *Global Health Sector Strategy on Viral Hepatitis*, *2016–2021* (GHSS) with the explicit goal of eliminating viral hepatitis “as a public health threat” by 2030 [9]. The core of GHSS relies on the so-called HCV cascade of care, which comprehensively summarizes the status of the HCV continuum of care in a particular setting, thereby acting as the key organizing framework for monitoring. It provides an overview of the status of the total number of estimated HCV cases: the number tested, diagnosed and confirmed, referred to specialist care, treated and cured [9]. On-site testing services at harm reduction centres, pre-test counselling and education, dried blood spot testing, facilitated referral for HCV assessment and scheduling of specialist appointments have proved useful in enhancing access to various stages of the cascade, including for vulnerable populations such as the homeless, the uninsured, PWID and other populations that may otherwise have no access to formal medical facilities [10, 11].

Injecting drug use is the main source of HCV infection in the European Union (EU), accounting for more than 80% of new infections [12]. PWID bear a significant burden of viral disease [13, 14], and the guidelines of the European Association for the Study of the Liver (EASL) recommend that they be evaluated for treatment [15]. Therefore, the governments are tasked by the WHO GHSS [9] with making comprehensive efforts to provide HCV-specific harm reduction services to PWID, which should be tailored to the continuum-of-care services for HCV [16–18]. Modelling studies conclude that a massive scale-up of testing and treatment is needed to reach the WHO elimination goals by 2030 [19]. However, fewer than half of the EU countries have national HCV strategies or action plans, and even fewer specifically address the needs of PWID in their hepatitis response documents. Moreover, the prevention, testing, linkage to care and treatment needs of HCV patients are far from being universally addressed in the EU [20, 21].

HCV is systematically underdiagnosed in Europe [22, 23], especially among PWID [24]. In addition, specific barriers hinder the inclusion of PWID and their linkage to the HCV cascade of care, and most PWID are largely unaware of their disease status [22–24] and of the availability of curative treatment regimens [25]. Some members of marginalized groups are so stigmatized that they hesitate to seek help [26, 27], and not all health systems are equipped to manage the complex social situations or health profiles of PWID. PWID are also deterred from seeking help from the authorities due to the criminalization of drug use, discrimination in healthcare settings and other violations of human rights [28]. As a result, few people living with HCV in Europe have received treatment and, until recently, drug use has been a common criterion for treatment ineligibility [29, 30].

The availability of services for the HCV cascade of care and national strategies for eliminating HCV has been identified as a key issue for the success of the WHO GHSS [31]. The aim of this study was to determine HCV service provision in harm reduction centres in all EU member states.

## Methods

Harm reduction services were defined as those activities and resources that encompassed opioid substitution therapy (OST), needle and syringe programmes (NSP) and the generic cross-cutting aspects of harm reduction [32]. These harm reduction services were referred to as being ‘free of charge’ if the client obtained them for free. HCV care was defined as activities that involved testing, linkage to care, counselling, treatment and surveillance of complications.

An online survey was developed and conducted by partners in the EU Joint Action on HIV and Co-infection Prevention and Harm Reduction (HA-REACT), with substantial input from the Correlation Network [33]. The survey was sent out on 29 March 2017 to relevant members of the Correlation Network providing or organizing harm reduction services in the 28 EU member states. Due to the unique health system structure of the UK and Spain, both of which are highly decentralized with subnational bodies managing the service, for the UK, surveys were sent to organizations in England and Scotland, and for Spain, they were sent to the Basque Country and Catalonia. Follow-up emails and calls were sent to participating organizations until responses from at least one organization in each of the EU member states were recorded. The survey was closed on 17 May 2017.

Simple frequency analysis was conducted of the replies to each survey question in each of the EU member states, for entities both organizing and providing harm reduction services. In cases where multiple responses were received from a country and the answers were concordant, this answer was reported as the overall response from the respective country. In cases where the responses were discordant between organizers and providers of harm reduction services, the response from the harm reduction service provider was accepted as the overall response from the country, since service providers were the main respondents. Where both parties were harm reduction service providers and the responses were discordant, the response was marked as inconclusive. These criteria were not applied to the UK or Spain, as the health systems of those countries necessitated that responses from different parts of the country be reported separately.

## Results

Thirty-eight harm reduction service providers from the 28 EU member states responded to the survey. Since England and Scotland reported separately as they have different healthcare systems, as is the case with the Basque Country and Catalonia in Spain, the total number of responses was 30. There were two responding harm reduction service entities from Belgium, Bulgaria, the Czech Republic, France, Lithuania, Poland and the UK. Three respondents were from Italy and Spain. The rest of the EU member states had only one respondent each.

### Testing

Twenty-three out of the 30 respondents (77%) reported that at least some harm reduction service providers offer HCV testing in their respective countries or regions. Five (17%; Croatia, Cyprus, Lithuania, Poland and Slovakia) reported that HCV testing is not available at any harm reduction service in their respective country or region, and two (7%; Basque Country and Slovenia) did not know (Table 1).

**Table 1:** EU member states in which hepatitis C tests are offered by any harm reduction service in the country and type of harm reduction services they offer (*N* = 30)

Hepatitis C tests are offered by any harm-reduction service in the country		Type of harm reduction services				
		Needle and syringe programmes	Opioid substitution therapy	Mental health services required to obtain opioid substitution therapy programmes	Other	Do not know
Austria	Yes					
Belgium	Yes	*	*	*		
Bulgaria	Yes	x			Not specified	
Croatia	No					
Cyprus	No					
Czech Republic	Yes	*	*	*		
Denmark	Yes					
Estonia	Yes					
Finland	Yes					
France	Yes					
Germany	Yes					
Greece	Yes					
Hungary	Yes	x	x			
Italy	Yes	x	x			
Ireland	Yes					
Latvia	Yes					
Lithuania	No					
Luxembourg	Yes	x	x			
Netherlands	Yes					
Malta	Yes					
Poland	No					
Portugal	Yes					
Romania	Yes					
Slovakia	No					
Slovenia	Do not know					
Spain (Barcelona)	Yes					
Spain (Bilbao)	Do not know					
Sweden	Yes					
UK (London)	Yes					
UK (Glasgow)	Yes					
Yes = 23 No = 5 Do not know = 2		*n* = 4/28	*n* = 3/28	*n* = 3/28	*n* = 1	*n* = 0

x: the respondent states that harm reduction services provide the HCV service in his/her country

*Answers from respondents differed, indicating that barriers may be dependent on the organization and differ within the country

#### Follow-up comments

- Cyprus: harm reduction service providers must refer patients to hospitals for treatment. However, training in rapid testing was expected to start in 2017, and staff working in harm reduction and treatment services will be able to provide HCV testing to Cypriot patients at some point between 2017 and 2020.
- Germany: even though harm reduction service providers can technically provide HCV testing, in practice, very few providers offer this service.

Twenty-six out of 29 respondents (90%; responses from Belgium were discordant and therefore removed from further analysis) reported that HCV testing is generally available free of charge in their respective countries or regions. Respondents from three countries (10%; Greece, Lithuania, Sweden) reported that HCV testing is generally not available free of charge in those countries.

### Care

Respondents from 26 of 30 (87%) countries or semi-autonomous regions reported that harm reduction service providers are involved in at least one aspect of managing HCV care. Two of the 30 (7%; Slovakia and Sweden) reported that harm reduction service providers are not involved in any aspect of managing HCV care, and two respondents (7%; Basque Country and Slovenia) did not know (Table 2).

**Table 2:** EU member states in which organizations that provide services to PWID are involved in managing any aspect of hepatitis C care (*N* = 30)

Country	Answer
Austria	Yes
Belgium	Yes
Bulgaria	Yes
Croatia	Yes
Cyprus	Yes
Czech Republic	Yes
Denmark	Yes
Estonia	Yes
Finland	Yes
France (Paris)	Yes
Germany	Yes
Greece	Yes
Hungary	Yes
Ireland	Yes
Italy (national)	Yes
Latvia	Yes
Lithuania (Vilnius)	Yes
Luxembourg	Yes
Malta	Yes
Netherlands	Yes
Poland (Krakow)	Yes
Portugal	Yes
Romania	Yes
Slovakia	No
Slovenia	Do not know
Spain (Barcelona)	Yes
Spain (Bilbao)	Do not know
Sweden	No
UK (London)	Yes
UK (Glasgow)	Yes
Total	Yes = 26 No = 2 Do not know = 2

#### Follow-up comments

- Belgium: clarified that there is a pilot “HCV Buddy Project” that helps guide patients through treatment and care.
- Czech Republic: added that harm reduction service providers offer case management to HCV patients.

Twenty-nine out of 30 respondents (97%) reported that NSP are generally available free of charge in their respective countries or regions. Only the respondent from Bulgaria reported that NSP were not available free of charge in that country (Table 3).

**Table 3:** Harm reduction and health services generally free of charge for PWID (*N* = 30)

	NSP	OST	Counselling	HIV testing	HCV testing	ART	HCV treatment
Austria	x	x	x	x	x	x	x
Belgium	x	*	x	x	*	*	*
Bulgaria				x	x	x	
Croatia	x	x	x	x	x	x	x
Cyprus	x	x	x	x	x	x	x
Czech Republic	x		x	x	x	x	x
Denmark*	x	x		x	x	x	x
Estonia	x	x	x	x	x	x	x
Finland	x	x	x	x	x	x	x
France	x	x	x	x	x	x	x
Germany	x	x	x	x	x	x	x
Greece	x	x		x		x	
Hungary	x	x	x	x	x	x	x
Italy	x	x	x	x	x	x	x
Ireland	x	x	x	x	x	x	x
Latvia	x	x	x	x	x	x	x
Lithuania	x	x	x			x	x
Luxembourg	x	x	x	x	x		
Netherlands	x	x	x	x	x	x	x
Malta	x	x	x	x	x	x	
Poland	x	x	x	x	x	x	x
Portugal	x	x	x	x	x	x	x
Romania	x	x	x	x	x	x	
Slovakia	x	x	x	x	x		
Slovenia	x	x	x	x	x	x	x
Spain (Barcelona)	x	x	x	x	x	x	x
Spain (Bilbao)	x	x	x	x	x	x	x
Sweden^+^	x		x	x		x	x
UK (London)	x	x	x	x	x	x	x
UK (Glasgow)	x	x	x	x	x	x	x
	*n* = 29	*n* = 26/29	*n* = 27	*n* = 29	*n* = 26/29	*n* = 27/29	*n* = 23/29

x: the respondent states that harm reduction services provide the HCV service in his/her country

*According to information shared by EMCDDA, Denmark has psychosocial counselling as part of treatment programmes (http://www.emcdda.europa.eu/countries/drug-reports/2017/denmark/treatment_en)

+According to information shared by EMCDDA, Sweden provides OST free of charge (http://www.emcdda.europa.eu/countries/drug-reports/2017/sweden/treatment_en)

Twenty-seven out of 29 respondents (93%; the responses from Belgium were discordant and therefore removed from analysis) reported that OST was available free of charge in their respective countries or regions. Respondents from three countries (10.3%; Bulgaria, Czech Republic and Sweden) reported that OST was not available free of charge in that country. The reply from Sweden showed a disagreement between the knowledge of national policies among stakeholders and the policies actually in place.

Respondents from 27 out of 30 (90%) countries reported that counselling was generally available free of charge in their respective countries or regions. Respondents from three countries (10%; Bulgaria, Denmark, Greece) reported that counselling was not available free of charge in those countries. The reply from Denmark showed a disagreement between the knowledge of health services among stakeholders and the services actually in place.

#### Follow-up comments

- Cyprus: clarified that care is generally free of charge at all government hospitals. For those clients who are not hospital card holders and are not citizens of Cyprus or the EU, there is a special system in place where the Ministry of Health can authorize free access to treatment. This is especially relevant for migrants from outside the EU.

### Treatment

Of the respondents in countries or semi-autonomous regions that had HCV tests available at harm reduction services (*n* = 27), 17 (63%) reported that their respective organizations had established referral systems with hospitals or clinics that provide HCV treatment (Table 4).

**Table 4:** EU member states in which there is an established referral system with a hospital or clinic that provides hepatitis C treatment (*N* = 27*)

Country	Answer
Austria	Yes
Belgium	Yes
Belgium	Yes
Bulgaria (Sofia)	No
Bulgaria (Burgas)	Yes
Czech Republic (Prague)	Yes
Czech Republic (Brno)	No
Denmark	No
Estonia	Yes
Finland	Yes
France (Paris)	Yes
France (Marseille)	Yes
Germany	Yes
Greece	No
Hungary	Yes
Italy (national)	No
Italy (Milan)	Yes
Ireland	Yes
Latvia	No
Luxembourg	Yes
Netherlands	No
Malta	Yes
Portugal	Yes
Romania	No
Spain (Barcelona)	No
Sweden	No
UK (London)	No
UK (Glasgow)	Yes
	Yes = 17 No = 10

*Respondents who reported that HCV testing is available in their countries

Eight out of 30 respondents (27%; Cyprus, the Czech Republic, Denmark, England, Germany, Luxembourg, Romania and Slovenia) reported that addiction specialists could prescribe HCV treatment in those countries or regions. Respondents from 20 of 30 (67%) countries or semi-autonomous regions reported that addiction specialists could not prescribe HCV treatment, and respondents from two countries (Ireland and Latvia) did not know (Table 5).

**Table 5:** EU member states in which addiction specialists can prescribe hepatitis C treatment (*N* = 30)

Country	Answer
Austria	No
Belgium	No
Bulgaria	No
Croatia	No
Cyprus	Yes
Czech Republic	Yes
Denmark	Yes
Estonia	No
Finland	No
France	No
Germany	Yes
Greece	No
Hungary	No
Italy	No
Ireland	Do not know
Latvia	Do not know
Lithuania	No
Luxembourg	Yes
Netherlands	No
Malta	No
Poland	No
Portugal	No
Romania	Yes
Slovakia	No
Slovenia	Yes
Spain (Barcelona)	No
Spain (Bilbao)	No
Sweden	No
UK (London)	Yes
UK (Glasgow)	No
	Yes = 8 No = 20 Do not know = 2

## Discussion

This study shows that at harm reduction services, the HCV cascade of care for PWID is severely constrained by a number of barriers in EU member states. This worrisome situation puts at risk the achievement of the WHO HCV elimination goals in the EU and entails a high burden of disease and financial consequences for the EU population as a whole (e.g. not only direct medical costs, often with highly expensive procedures such as liver transplantation, but also indirect costs as those due to decreased work productivity) [34–38]. Health authorities at the EU and country levels should therefore take measures to overcome the challenges of eliminating HCV among PWID in a way that preserves human rights and mitigates health inequalities [39].

### Testing

HCV tests are offered by harm reduction service providers in the majority of EU member states, except in about one fifth of the countries and semi-autonomous regions surveyed. Respondents also indicated that HCV testing was not free of charge in three EU member states.

The Hep-CORE study conducted by the European Liver Patients’ Association was similar to ours but included responses from seven non-EU countries [20]. It showed that free HCV testing was available in one third of the countries in 2016. As EU member states are generally wealthier and have larger budgets for healthcare services than their European non-EU counterparts, non-EU countries could be less likely to have free HCV testing available than EU countries. According to the Hep-CORE study, patient groups also reported that about half of their respective countries had testing available outside of hospitals for the general population. This figure was also significantly higher in our survey.

According to a 2016 data from the EMCDDA, only 47.4% of the countries assessed had over 50% OST coverage for high-risk opioid users and just 26.7% had high coverage of specialized NSP [40]. This shortage of harm reduction services is a major barrier to connecting PWID to testing [41, 42], as facilities providing harm reduction services are usually in a better position than healthcare settings to contact and test members of the community. Moreover, the fact that not all EU countries have testing available at harm reduction services indicates a missed opportunity. Policies regarding HCV testing should be amended to solve this problem, including the implementation of point-of-care testing to improve the coverage of diagnosis, among others [43]. It has further been shown that PWID are more likely to be tested for HCV if they have access to point-of-care HCV antibody testing rather than testing at traditional healthcare sites [44]. Moreover, there is evidence that screening is cost effective and helps lessen the financial burden of HCV on health systems [45].

### Care

Respondents in our survey reported that harm reduction service providers in two countries or regions were not involved in managing any aspect of HCV care. Perhaps the most striking finding was that respondents from two thirds of the EU member states or regions reported that addiction specialists could not prescribe HCV treatment, in spite of PWID being one of the highest burden HCV-infected populations in Europe. These results suggest that targeted efforts are needed to expand on-site testing services at harm reduction facilities in Croatia, Cyprus, Lithuania, Poland and Slovakia, and potentially in Slovenia and Spain’s Basque Country as well. Although pre-test counselling and dried blood spot testing were not a focus of our survey, these services should be included in all existing and future testing services of harm reduction service providers in the EU.

### Treatment

Despite the existence of an effective biomedical cure, the socioeconomic and health system realities of the disease currently hamper the possibility of global HCV elimination. Reimbursement restrictions and the high prices of direct-acting antivirals (DAAs) limit their accessibility, even in high-income countries [46, 47]. DAAs are effective in achieving a sustained virological response in patients, effectively curing them [15]. PWID attending harm reduction services can achieve the same level of adherence as the general population [48, 49], including when treatment is provided at the community level [50].

Our study shows that addiction specialists are able to prescribe DAAs only in a handful of EU member states. The issue of whether addiction medicine specialists should be allowed to prescribe DAAs to widen the scope of treatment, or whether linkage to care from harm reduction centres to infectious disease specialists and hepatologists should be improved has generated a controversy. Pivotal studies performed in Australia have provided valuable insight pointing to the feasibility, cost efficacy and lower rates of follow-up losses when properly educated, and trained addiction doctors are directly involved in HCV treatment [51, 52]. From our perspective, this approach needs to guarantee that the HCV cascade of care is available to PWID so as to achieve the WHO elimination goals.

Previous studies have shown that financial barriers prevented HCV patients in Eastern EU member states from accessing HCV treatment in the era of interferon-based therapies [53]. In Romania, for example, the high costs of treatment resulted in 89% of patients diagnosed with chronic HCV forgoing treatment [54]. In Romania, the reimbursement scheme does not seem to have changed, as the respondent from Romania in the present survey said that HCV treatment is not provided to patients free of charge.

### Limitations

The greatest limitation of our study is that it surveyed only harm reduction service providers, who are not necessarily able to report on national health policy and governance. Therefore, there may be discrepancies between harm reduction providers’ perception or understanding of policy, and the policy as it is written—such as the cases highlighted from Sweden and Denmark. Further, as many of the questions in the survey were not open-ended, the complexities of policy as it plays out in practice may have been lost. Even if DAA treatment is available, there are restrictions in prescribing them [20]. Our study did not review any such restrictions, and therefore some of the countries where we report that DAAs are available may not provide these to all HCV patients.

In addition, our survey does not report the availability of dried blood spot testing, although this has been shown to be a simpler diagnostic tool [10].

## Conclusions

Eliminating hepatitis C as a public health threat requires a concerted effort targeting PWID. This study shows that, according to providers, not all EU member states have harm reduction services that are suited for this purpose. The services available were reported to lack both HCV testing and an established referral system to HCV treatment providers. Furthermore, in many countries, addiction specialists are not authorized to prescribe HCV treatment. Finally, discrepancies were noted between national policies in place and harm reduction stakeholders’ knowledge of and information about these policies, indicating a gap between policy and practice.

The way forward requires a multifaceted response involving at least the following aspects. First, HCV tests should be available at all harm reduction facilities. Second, current prescribing restrictions should be overcome, and addiction doctors should be able to provide HCV treatment to optimize its accessibility. Third, HCV medication should be made free of charge for patients. And fourth, practice and policy should be reviewed at the national and sub-national levels, and stakeholders should be involved to a greater degree in the development of policies to ensure better implementation. Improving prevention, treatment and care for PWID is in line with the United Nations comprehensive package’s recommendation that PWID be diagnosed and treated for viral hepatitis.

## Abbreviations

DAA: Direct-acting antiviral
ECDC: European Centre for Disease Prevention and Control
EMCDDA: European Monitoring Centre for Drugs and Drug Addiction
EU: European Union
GHSS: Global health sector strategy on viral hepatitis 2016–2021
HA-REACT: EU Joint Action on HIV and Co-infection Prevention and Harm Reduction
HCV: Hepatitis C virus
MSM: Men who have sex with men
NSP: Needle–syringe programme
OST: Opioid substitution therapy
PWID: People who inject drugs
UN: United Nations
WHO: World Health Organization
